# Determination of organically bound fluorine sum parameters in river water samples—comparison of combustion ion chromatography (CIC) and high resolution-continuum source-graphite furnace molecular absorption spectrometry (HR-CS-GFMAS)

**DOI:** 10.1007/s00216-020-03010-y

**Published:** 2020-11-08

**Authors:** Lennart Gehrenkemper, Fabian Simon, Philipp Roesch, Emily Fischer, Marcus von der Au, Jens Pfeifer, Antje Cossmer, Philipp Wittwer, Christian Vogel, Franz-Georg Simon, Björn Meermann

**Affiliations:** 1 Division 1.1 - Inorganic Trace Analysis, Federal Institute for Materials Research and Testing (BAM), Richard-Willstätter-Straße 11, 12489 Berlin, Germany; 2 Division 4.3 - Contaminant Transport and Environmental Technologies, Federal Institute for Materials Research and Testing (BAM), Unter den Eichen 87, 12205 Berlin, Germany; 3 Department G2 - Aquatic Chemistry, Federal Institute of Hydrology (BfG), Am Mainzer Tor 1, 56068 Koblenz, Germany

**Keywords:** High resolution-continuum source-graphite furnace molecular absorption spectrometry (HR-CS-GFMAS), Combustion ion chromatography (CIC), Per- and polyfluorinated alkyl substances (PFASs), Adsorbable organically bound fluorine (AOF), Extractable organically bound fluorine (EOF), Surface waters

## Abstract

In this study, we compare combustion ion chromatography (CIC) and high resolution-continuum source-graphite furnace molecular absorption spectrometry (HR-CS-GFMAS) with respect to their applicability for determining organically bound fluorine sum parameters. Extractable (EOF) and adsorbable (AOF) organically bound fluorine as well as total fluorine (TF) were measured in samples from river Spree in Berlin, Germany, to reveal the advantages and disadvantages of the two techniques used as well as the two established fluorine sum parameters AOF and EOF. TF concentrations determined via HR-CS-GFMAS and CIC were comparable between 148 and 270 μg/L. On average, AOF concentrations were higher than EOF concentrations, with AOF making up 0.14–0.81% of TF (determined using CIC) and EOF 0.04–0.28% of TF (determined using HR-CS-GFMAS). The results obtained by the two independent methods were in good agreement. It turned out that HR-CS-GFMAS is a more sensitive and precise method for fluorine analysis compared to CIC. EOF and AOF are comparable tools in risk evaluation for the emerging pollutants per- and polyfluorinated alkyl substances; however, EOF is much faster to conduct.

**Figure:**
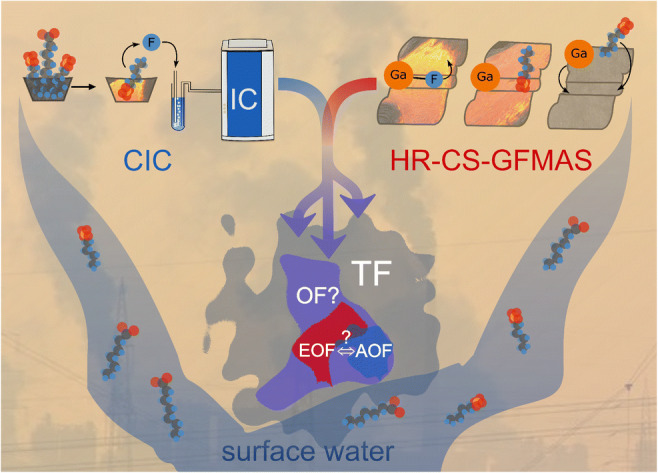
Graphical abstract

Graphical abstract

## Introduction

Substitution of hydrogen with fluorine in organic molecules affects their chemical and physical properties, e.g., increased chemical and thermal stability and/or oleophobic as well as hydrophobic properties [1, 2]. These molecules are described according to Buck et al. as per- and polyfluorinated alkyl substances (PFASs) [3]. PFASs are organic compounds in which all the hydrogen atoms on at least one carbon atom are replaced by fluorine [3]. Highly fluorinated organic substances are used as surfactants in technical applications, e.g., as water and grease protection agents in carpets [4], clothing [5], and food packaging [6], and as fire-extinguishing agents [7]. During production, use, and disposal of these industrial products, PFASs enter the environment. Because of their extreme persistence, PFASs accumulate in the abiotic environment, e.g., ground [8] and surface water [9], soil [10], and air [11], as well as in the biotic environment [12, 13]. Overall, there are large amounts of human exposure pathways so that PFASs can be detected in human serum [14, 15] as well as in human breast milk [16]. The concerning notice that PFASs can be found ubiquitously, even in the arctic environment [17], and that there are potential negative effects on the environment and human health [18] lead to first limitations. Since 2009, perfluorooctanesulfonic acid (PFOS) and, since 2019, perfluorooctanoic acid (PFOA) are listed in annexes of the Stockholm Convention on Persistent Organic Pollutants [19]. For these reasons, the production and use of PFOS and PFOA must be reduced respectively avoided [19]. Since then, PFOS and PFOA are substituted with shorter- (≤ 6 perfluorinated carbons [20]) and longer-chained (≥ 7 perfluorinated carbons [20]) PFASs, which aren’t potentially less persistent or risky [21]. The huge amount of different PFASs (> 4700 [22]) and this replacement lead to new analytical challenges. Because of their extreme persistence and vast anthropogenic emission, PFASs are emerging pollutants.

In this context, the environmental compartment water is particularly interesting. Especially through the ineffective removal of PFASs by conventional treatments of waste water treatment plants (WWTP) [23], PFASs accumulate in the aquatic environment and lead to contamination of ground and drinking water, thus entering the food chain (e.g., plants [24], animals [25, 26], and humans [27]).

PFASs target analytic in aqueous samples by liquid chromatography coupled with mass spectrometry (LC-MS) and gas chromatography coupled with mass spectrometry (GC-MS) methods cover only a small proportion (7–53 PFASs for LC-MS [28–30], and 10–13 PFASs for GC-MS [31, 32]) of the over 4700 different PFASs and vastly underestimate the quality and quantity of total organically bound fluorine (OF) [33]. This results in a huge gap in the PFAS mass balance with an unknown amount of potentially toxic and persistent PFASs. Consequently, a sum parameter method for organically bound fluorine is inevitable to cover the unknown proportion of PFASs.

PFASs in aqueous samples can be extracted using a sorbent (adsorbable organically bound fluorine, AOF) or alternatively using an organic solvent (extractable organically bound fluorine, EOF) [34]. Usually activated carbon (AC) is used as sorbent for AOF determination. Hence, AOF represents all PFASs present in water samples which are adsorbable on AC. Which PFASs are summed up in the EOF depends on the solid-phase material used during the extraction. Often, a weak anion exchanger (WAX) is used. Using WAX-solid-phase extraction (SPE), the EOF covers only neutral and anionic PFASs. In the literature, there is also the use of hydrophilic-lipophilic balance (HLB) materials described, resulting in a wider range of extracted PFASs [9].

The sample preparation for the determination of AOF is carried out according to the standardized adsorbable organically bound halogen (AOX) method ISO 9562 (adsorption on AC and argentometric determination) [35]. Based on ISO 9562, Wagner et al. developed a combustion ion chromatography (CIC) method, applicable to determine the AOF as well. Upon adsorption, AC is combusted and fluorine is converted into hydrogen fluoride (HF), which is then adsorbed in a trapping solution. Subsequently, the analysis of fluoride was carried out using ion chromatography (IC) [36]. A complementary target analysis by Willach et al. connoted that the AOF of some highly contaminated aqueous samples can only be explained by < 5% with LC-MS approaches, which underlines the importance of an organically bound fluorine sum parameter [33].

The determination of EOF in aqueous samples was firstly described by Miyake et al. in 2007. For sample preparation, they used an SPE method comprising a WAX phase. The eluent was measured via CIC in accordance with the aforementioned AOF CIC approach. With a different analytical approach utilizing high resolution-continuum source-graphite furnace molecular absorption spectrometry (HR-CS-GFMAS), Metzger et al. developed a method for the determination of EOF using in situ formation of GaF in the graphite furnace for detection. GaF is the most sensitive diatomic molecule for fluorine analysis using HR-CS-GFMAS in surface water analysis [37]. In contrast to the previously developed method by Miyake et al. using WAX as SPE material, Metzger et al. used an HLB material for SPE.

**Fig. 1:**
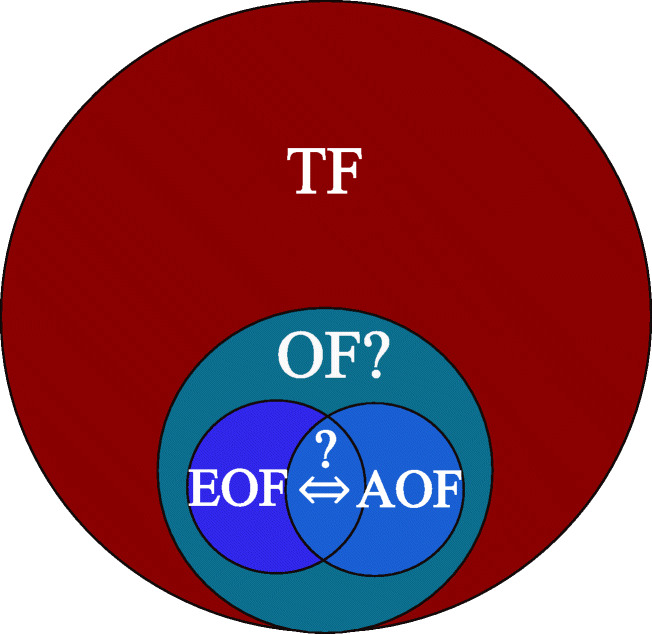
Scheme of a fluorine mass balance approach applying organically bound fluorine sum parameters

The overall aim of this study is the comparison of fluorine analysis using either CIC or HR-CS-GFMAS. Additionally, a mass balance and sum parameter analysis of OF is applied, which is schematically described in Fig. 1. This approach involves the determination of TF, followed by the complementary adsorption respectively extraction of organic fluorine. By comparing the concentration and composition of EOF/AOF, it can be estimated, which sum parameter reflects OF better and which sum parameter is therefore advantageous in risk evaluation and understanding of the environmental prevalence of the emerging pollutant PFASs. Coherently, the accurate and sensitive determination of these sum parameters using either CIC or HR-CS-GFMAS plays an equally important role for risk evaluation. Revealing the most advantageous sum parameter for organically bound fluorine with the most sensitive analytical method is therefore the aim of this study.

## Materials and methods

### Chemicals

Deionized water was produced using a Milli-Q® Advantage A10 System (Merck KGaA, Darmstadt, Germany) for HR-CS-GFMAS experiments (at Federal Institute for Materials Research and Testing (BAM), HR-CS-GFMAS_B_), and for CIC experiments at the Federal Institute of Hydrology (BfG; instrumental setup in the following abbreviated as CIC_KO_); a LaboStar DI 2 system (Siemens Evoqua Water Technologies GmbH, Günzburg, Germany) generating ultrapure water for CIC experiments at the Federal Institute for Materials Research and Testing (BAM; instrumental setup in the following abbreviated as CIC_B_). Nitric acid (65%, p.a.) was purchased from ChemSolute® (Th. Geyer GmbH & Co. KG, Renningen, Germany) and subboiled prior use via a two-stage custom made subboiling system (PicoTrace Subboiling Kuppel-Destille SCD, PicoTrace GmbH, Göttingen, Germany). Zirconium, calcium, magnesium, and palladium solutions (all Certipur® grade purchased from Merck KGaA, Darmstadt, Germany) were used as HR-CS-GFMAS_B_ modifiers and PIN platform (Analytik Jena AG, Jena, Germany) coating reagents respectively. Aqueous sodium acetate solution 10 g/L as HR-CS-GFMAS_B_ modifier was prepared from sodium acetate trihydrate (BioChemica grade, AppliChem GmbH, Darmstadt, Germany). Gallium(III) nitrate hydrate (trace metals basis, 99.999%) was purchased from Sigma-Aldrich. Fluoride standard solution and ortho-phosphate standard solution were obtained from Merck (all 1000 mg/L; Certipur®, Merck KGaA, Darmstadt, Germany). Methanol was purchased from Merck (hypergrade for LC-MS; LiChrosolv®, Merck KGaA, Darmstadt, Germany). Sodium carbonate and sodium hydrogen carbonate stock solutions were obtained from Thermo Fisher Scientific (0.5 M, Thermo Fisher Scientific GmbH, Dreieich, Germany). Activated carbon was purchased from Analytik Jena (50–150 μm, from Analytik Jena AG, Jena, Germany). 4-Fluorobenzoic acid (99%; purified by sublimation) was purchased from Merck (Merck KGaA, Darmstadt, Germany). Sodium nitrate (99.5%) was purchased from ChemSolute® (Th. Geyer GmbH & Co. KG, Renningen, Germany). Ammonium fluoride (p.a.) was obtained from Merck (Supelco®, Merck KGaA, Darmstadt, Germany). Ammonium solution (25%) was purchased from Merck (Suprapur®, Merck KGaA, Darmstadt, Germany). For determination of accuracy during HR-CS-GFMAS_B_ measurement, the following fluoride-containing-certified reference material (CRM) was used: ION-96.4 environmental matrix reference material (c(F) = 0.123 ± 0.034 mg/L, Environment and Climate Change Canada, Canada).

### Sampling

**Fig. 2:**
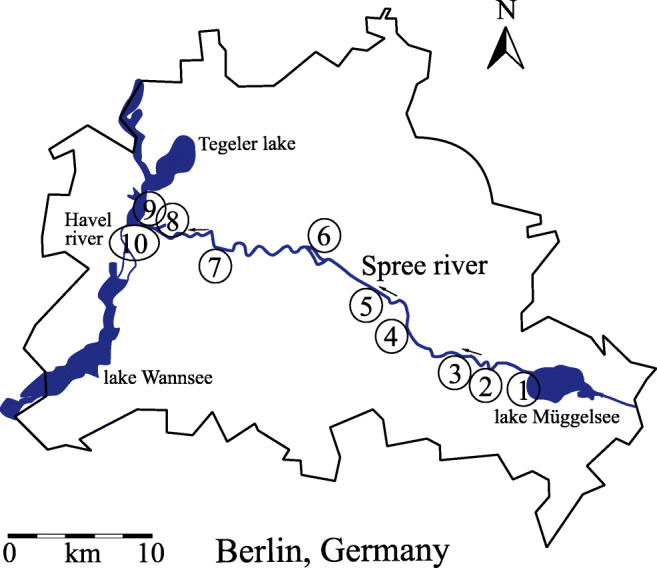
Sampling locations along the river Spree in Berlin

Water samples from the river Spree were taken on 4^th^ of June 2020 on ten spots along its way through Berlin, Germany. Coordinates of the sampling locations were tracked using a GPSMAP® 64SX (Garmin Ltd., Olathe, USA) and are listed in Table 1. A map of the sampling locations is shown in Fig. 2. Each sample was collected at 20–30 cm depth under the water surface and 1.5–2.0 m distance to the riverbank using a leached sample bottle (LDPE high performance bottles, VWR, Darmstadt, Germany) mounted on a telescope pole. Sample bottles were conditioned with river water before filling. On each spot, 6 samples of 500 mL were collected. Water temperature, conductivity, pH value, and O_2_ concentration were measured in a separate vessel using a Multi 3430 Set G (Wissenschaftlich-Technische Werkstätten, Weilheim, Germany). Measured environmental parameters for each location are summarized in Table 1. Water samples and two blanks (deionized water) were filtered on the day of sample collection using nitro cellulose membrane filters with a pore size of 0.45 μm (LABSOLUTE®, Th. Geyer GmbH & Co. KG, Renningen, Germany) and stored in a refrigerator at 4 °C in the dark to reduce the potential growth of microorganisms.

**Table 1:** Water parameters measured during sample collection and sampling location coordinates

Sample	Coordinates	pH	*T* (°C)	*λ* (μS/cm)	*c*(O_2_) (mg/L)
1	N52°26.656′	7.99	19.3	838	8.95
	E013°37.376′				
2	N 52°26.928′	7.74	21.5	870	7.19
	E 013°34.152′				
3	N 52°27.190′	7.67	20.0	828	6.96
	E 013°33.324′				
4	N 52° 28.324′	7.73	21.3	835	7.30
	E 013°29.712′				
5	N 52°29.504′	7.98	21.2	852	8.64
	E 013°28.224′				
6	N 52°31.168′	7.73	21.8	870	7.75
	E 013°24.142′				
7	N 52°31.236′	7.60	20.7	908	7.27
	E 013°18.346′				
8	N 52°31.998′	7.82	20.7	923	7.86
	E 013°14.122′				
9	N 52°32.088′	7.68	20.9	999	8.20
	E 013°13.650′				
10	N 52°32.113′	7.54	21.1	920	7.92
	E 013°12.434′

### Total fluorine analysis

To determine the amount of total fluorine (TF), 1 mL of each filtrated sample was directly analyzed in triplicate by means of contrAA 800 HR-CS-GFMAS system (Analytik Jena AG, Jena, Germany) and the software ASpect CS 2.2.2.0 (Analytik Jena AG, Jena, Germany). HR-CS-GFMAS_B_ measurements were performed following a protocol of Metzger et al. [9]. Zirconium-coated graphite furnaces with PIN platforms were prepared and conditioned as described previously [9]. Absorption of GaF formed in situ in the graphite furnace was measured at a wavelength of 211.248 nm. Injection of the sample as well as modifiers was conducted as follows: 2 μL deionized water, 16 μL sample, 9 μL 1 g/L gallium solution, 3 μL 10 g/L sodium acetate solution, 3 μL modifier mix (consisting of 0.1% (v/v) of palladium, 0.05% (v/v) of magnesium matrix modifier, and 20 mg/L zirconium standard) and 2 μL deionized water. For quantification, an external calibration of aqueous fluoride standard with concentrations of 0, 40, 80, 120, 160, 180, 200, 220, and 250 μg/L was used. To prevent enrichment of the analytes through evaporation of solvents, each sample vessel was covered with Parafilm® M purchased from Th. Geyer (Th. Geyer GmbH & Co. KG, Renningen, Germany). Samples were measured in instrumental triplicates.

Additionally, TF analysis was carried out using CIC_B_ consisting of a combustion system (AQF-2100H, Mitsubishi Chemical, Tokyo, Japan) and an IC (ICS Integrion, Thermo Fisher Scientific GmbH, Dreieich, Germany) controlled by the software Chromeleon 7.2.10 (Thermo Fisher Scientific GmbH, Dreieich, Germany). The combustion unit consisted of an autosampler (ASC-210) connected to the induction furnace unit (AQF-2100H) maintained at constant 1050 °C operated by the NSX 2100 software (instrumental setup is summarized in Table 2 and Table 3). Before use, all ceramic boats were thermolytically cleaned for at least 15 min at 1050 °C to avoid organic contamination. A liquid sample (0.5 mL) was loaded on a ceramic boat with a pipet (Transferpette, Brand GmbH + CO KG, Wertheim, Germany) and investigated via CIC_B_. Hydropyrolysis during combustion was enabled by a constant flow of dry O_2_ (300 mL/min) and water supplied argon (150 mL/min). Combustion gases were absorbed in 5 mL of a freshly prepared 0.1 mM NH_3_ absorption solution within the gas absorption unit (GA-210). Ion chromatography was performed using Dionex IonPac AG22 guard column (2 × 50 mm) as guard column and Dionex IonPac AS22 (2 × 250 mm) as analytic column (column temperature 30 °C), operated with an eluent consisting of 4.5 mM Na_2_CO_3_ and 1.4 mM NaHCO_3_ and flow rate of 0.3 mL/min. Fluoride ions were detected by a conductivity detector using 250 mM H_3_PO_4_ as suppressor regenerant. For calculation of detected peak areas and fluoride concentrations, the chromatography data system Chromeleon 7.2.10 was used. A seventeen-point standard calibration curve was prepared at 0.002, 0.004, 0.006, 0.008, 0.01, 0.02, 0.04, 0.06, 0.08, 0.1, 0.2, 0.4, 0.6, 0.8, 1.0, 2.0, and 5.0 mg/L fluoride using stock NH_4_F solutions (c(F) = 1000 mg/L).

**Table 2:** Combustion parameters for TF and EOF determination using CIC_B_

Combustion	
Combustion device	AQF-2100H, Mitsubishi Chemical Analytech Co., Ltd.
Operating temperature	1050 °C
Carrier gas flow	150 mL/min
Ar flow water supply	100 mL/min
O_2_ flow	300 mL/min
Absorption solution	1.0 mM NH_3_ solution + 200 μg/L NaH_2_PO_4_
Absorption volume	5 mL (TF); 4 mL (EOF)
Sample amount	500 μL (TF); 200 μL (EOF)
Water supply level	2

**Table 3:** Ion chromatography parameters for TF and EOF determination using CIC_B_

Ion chromatography	
IC device	ICS Integrion, Thermo Fisher Scientific
Detector	Conductivity detector
Guard column	AG22 2 × 50 mm guard column
Analytical column	Dionex IonPac AS22 2 × 250 mm
Eluent	4.5 mM Na_2_CO_3_/1.4 mM NaHCO_3_
Flow rate	0.3 mL/min
Run time	15 min
Column temperature	30 °C
Injection volume	100 μL
Suppressor regenerant	50 mM H_2_SO_4_

### Solid-phase extraction of extractable organically bound fluorine

SPE was carried out following the optimized protocol of Metzger et al. [9] and was done in triplicate for each sample and in duplicate for deionized water as blanks. Therefore, HLB-SPE cartridges (OASIS®, Waters, Eschborn, Germany) and a vacuum chamber (HyperSep™, Thermo Fisher Scientific GmbH, Schwerte, Germany) were used. The SPE cartridges were rinsed with 3.0 mL methanol and twice with 3.0 mL of an acidified aqueous solution (deionized water acidified with HNO_3_ to pH 2). The valves were closed, and the solid phases were covered with 2.5 mL of acidified aqueous solution (pH 2). Before the extraction step, the pH value of each filtrated sample was adjusted to pH 2 using HNO_3_. 250 mL of each sample was vacuumed through the cartridges; the solid phase was rinsed two times with 3.0 mL of the acidified aqueous solution (pH 2) and vacuum dried for 30 min. The extracted compounds were eluted by means of 1 mL methanol. Eluates were then evaporated to dryness in a vacuum spin evaporator system (RVC 2-25 CDplus, Christ Martin Gefriertrocknungsanlagen GmbH, Osterode am Harz, Germany) and stored until further analysis in a refrigerator at 4 °C in the dark. Before the measurement using HR-CS-GFMAS_B_ as described above (see Total fluorine analysis), samples were re-dissolved in 1 mL of methanol/water (1:1; v/v). For EOF calibration, a mixture of methanol/water (1:1; v/v) was used as solvent, resulting in concentrations at 0, 5, 10, 20, 40, 60, 80, 100, 120, 160, 200, and 250 μg/L fluoride.

Combustion ion chromatography analysis was conducted at the Federal Institute for Materials Research and Testing (CIC_B_). Therefore, 0.2 mL aliquots of the re-dissolved EOF samples were loaded on quartz wool (0.2 g)-packed ceramic boats, carefully evaporated prior to combustion and combustion gases absorbed in 4 mL of a freshly prepared 0.1 mM NH_3_ absorption solution within the gas absorption unit (GA-210). The same calibration curve was used as for TF determination.

### Adsorption and combustion of organic bound fluorine

Determination of the AOF in river water samples was divided in three steps, following a modified protocol of ISO 9562:2004-09: (i) adsorption of the organic fluorine on AC columns, (ii) combustion of the AC and absorption of released hydrogen fluoride in a trapping solution, and (iii) quantitative measurement of fluoride in the trapping solution using both IC and HR-CS-GFMAS.

(i) For the enrichment step, the pH value of each filtrated river water sample and each methodic blank, consisting of deionized water, was adjusted to pH 2 using HNO_3_. Samples were prepared as triplicates. Aliquots of 100 mL were automatically vacuum pumped through triplex quartz containers (Analytik Jena AG, Jena, Germany) packed with two times 55–60 mg AC (50–150 μm, from Analytik Jena AG, Jena, Germany) and once with a cotton pellet (4.0 mm, Orbis Dental Handelsgesellschaft mbH, Münster, Germany). The adsorption columns were washed with 25 mL of an aqueous sodium nitrate solution (c(NaNO_3_) = 0.01 mol/L) to remove ionic fluoride.
(ii) For combustion, the AC was transferred quantitatively into ceramic sample boats and hydropyrolyzed in a combustion device (AQF-2100H, A1 Enviroscience GmbH, Düsseldorf, Germany) at 1000 °C (CIC_KO_). During the combustion process, a carrier gas flow of 200 mL/min (Ar) and oxygen gas flow of 400 mL/min were applied. Additionally, an argon stream-supported water supply (100 mL/min) was used according to Wagner et al. [36]. CIC_KO_ combustion parameters are summarized in Table 4. During combustion, the adsorbed organically bound fluorine compounds were converted into HF, which was trapped in 10 mL of an aqueous phosphate solution (5 mg/L).
(iii) The trapping solution was split for analysis via IC and HR-CS-GFMAS_B_.

**Table 4:** Combustion parameters for AOF determination using CIC_KO_

Component	Parameter
Combustion device	A1 Enviroscience
Operating temperature	1000 °C
Carrier gas flow	200 mL/min
Ar flow water supply	100 mL/min
O_2_ flow	400 mL/min
Absorption solution	Phosphate solution (5 mg/L)
Absorption volume	10 mL
Sample amount	55–60 mg AC
Water supply level	2

(i) and (ii) steps of AOF analysis were conducted at the BfG in Koblenz, Germany. One set of trapping solutions out of the sample triplicates was shipped to Berlin by overnight express in cooled boxes and stored immediately in a refrigerator at 4 °C in the dark to be measured by means of HR-CS-GFMAS_B_ as described above (see Total fluorine analysis). HR-CS-GFMAS_B_ calibration solutions for AOF measurements contained additionally 5 mg/L phosphate, prepared by dilution of an ortho-phosphate standard solution to match the matrix of the trapping solution, resulting in concentrations at 0, 1, 2, 5, 10, 15, 20, 30, 40, 60, 80, and 100 μg/L fluoride.

For IC analysis of the AOF trapping solutions at the BfG, an 881 compact IC pro system (Metrohm GmbH & Co. KG, Filderstadt, Germany) equipped with a conductivity detector was used. IC was performed using Metrosep A Supp 5 Guard/4.0 as guard column and Metrosep A Supp 5 250/4.0 as analytical column (column temperature 45 °C), operated with an eluent consisting of 3.2 mM Na_2_CO_3_ and 1.0 mM NaHCO_3_. The flow rate was 0.7 mL/min. Parameters of the method are summarized in Table 5. For external calibration and quantification of fluoride, 4-fluorobenzoic acid purchased from Merck (Merck KGaA, Darmstadt, Germany) was dissolved in methanol. Calibration solutions were prepared by dilution of the 4-fluorobenzoic acid solution with deionized water to end up with the following calibration curve: 0, 4, 6, 8, 10, 20, 40, 60, 80, 100, 200, 400, 600, 800, and 1000 μg/L fluoride.

**Table 5:** Ion chromatography parameters for AOF determination using IC_KO_

Component	Parameter
IC device	881 compact IC pro, Metrohm
Detector	Conductivity detector
Pre column	Metrosep A Supp 5 Guard/4.0
Analytical column	Metrosep A Supp 5 250/4.0
Eluent	3.2 mM Na_2_CO_3_ and 1.0 mM NaHCO_3_
Flow rate	0.7 mL/min
Run time	40 min
Temperature	45 °C
Injection volume	100 μL
Suppressor regenerant	250 mM H_3_PO_4_

### Limit of detection/limit of quantification

Instrumental limit of detection (LOD) and limit of quantification (LOQ) were determined for fluorine analysis using HR-CS-GFMAS_B_ and CIC. Calculation was conducted according to DIN 32645 [38]. Therefore, 10 blank measurements (deionized water) and a calibration of the same day were taken. Subsequently, blank standard deviation (SD) was calculated, divided by the slope of the calibration curve, and multiplied by 3, resulting in the instrumental LOD value. Factor 10 was used for the determination of the instrumental LOQ.

### Data analysis

All data plots were created using Origin^®^2020 software (OriginLab Corporation, Northampton (MA), USA). Linear regressions and confidence intervals for the scatter plot (see Comparison of AOF determined via CIC and EOF determined via HR-CS-GFMAS, Results and discussion section) were calculated also using Origin^®^2020. (Relative-)Standard deviations and mean values were calculated using Microsoft Excel (Office 365 ProPlus, Redmond (WA), USA).

**Fig. 3:**
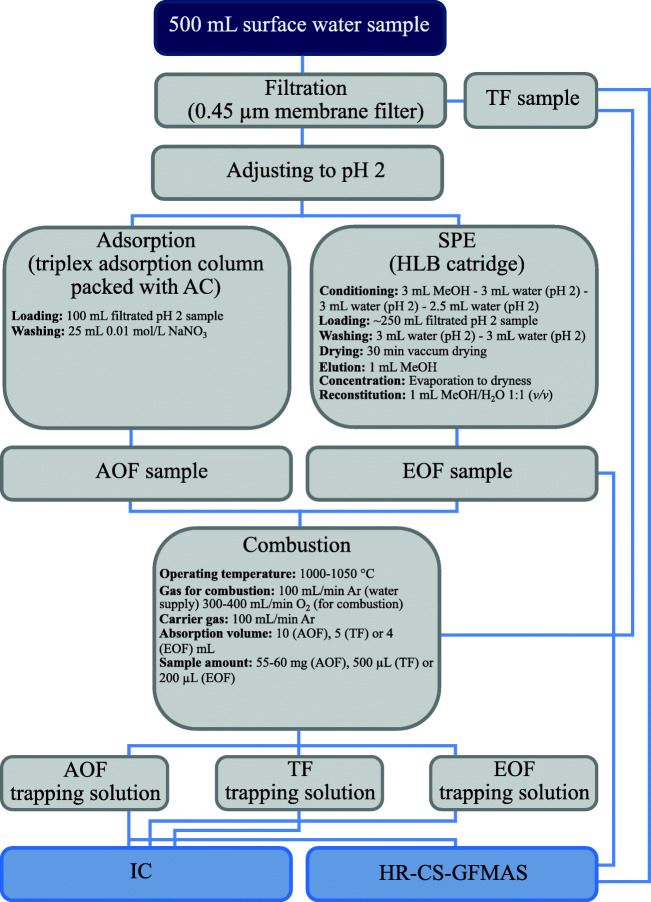
Sample pretreatment and analysis scheme

Figure 3 displays the sample pretreatment and analysis scheme deployed within this study.

## Results and discussion

### Determination of LOD and LOQ

Results for LOD and LOQ are shown in Table 6. HR-CS-GFMAS_B_ LOQ was the lowest with 2.7 μg/L, while CIC LOQs were around 10 μg/L. TF, EOF, and AOF concentrations mostly exceeded the LOQ of all instruments. Only one EOF sample (sample location 8) out of a triplicate was below CIC_B_ LOQ and ten AOF samples (sample locations 1, 2, 3, 4, 6, and 10) out of the triplicates were below the CIC_KO_ LOQ. Thus, according to the obtained LOD and LOQ, all instrumental methods are suitable for TF and EOF determination. For the analysis of AOF via CIC, a higher concentration factor should be chosen for quantitative measurements.

**Table 6:** Instrumental LOD and LOQ values for fluorine analysis using HR-CS-GFMAS_B_ and CIC (determined using DIN 32645)

	HR-CS-GFMAS_B_ (μg/L)	CIC_B_ (μg/L)	CIC_KO_ (μg/L)
LOD	0.8	3.2	3.0
LOQ	2.7	10.7	10.0

### Total fluorine analysis

**Fig. 4:**
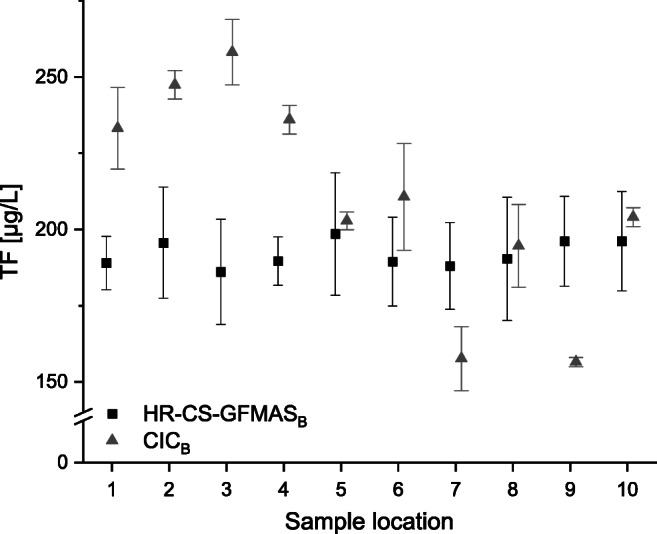
Total fluorine concentrations in Spree river water samples determined using HR-CS-GFMAS_B_ (square) and CIC_B_ (triangles). Error bars are based on *n* = 3 ± SD (methodical triplicate). The mean CRM fluorine concentration was 146.31 μg/L (acceptable according to the manufacturer). For sample locations, refer to Fig. 2 and Table 1

TF was determined using HR-CS-GFMAS_B_ and CIC_B_ and results are shown in Fig. 4. Samples were measured in technical and methodical triplicates. For HR-CS-GFMAS_B_, mean concentrations varied around ~ 190 μg/L with maximum concentrations of 213.5 μg/L at sampling location 8 and minimum concentration of 169.8 μg/L at location 3. Relative SD of three independent samples was between 4.2 and 10.6%. For CIC_B_, mean concentrations varied around ~ 210 μg/L with maximum concentrations of 269.8 μg/L at sampling location 3 and minimum concentration of 147.6 μg/L at location 9. Relative SD was between 0.9 and 8.3%. Similar concentration ranges were published for fluoride by Berliner Wasserbetriebe along the river Spree in Berlin [39]. On average, CIC_B_ concentrations for TF were ~ 20 μg/L higher than using HR-CS-GFMAS_B_. Additionally, relative SD was lower during CIC_B_ TF determination compared with HR-CS-GFMAS_B_. As mentioned above, TF concentration mostly depends on inorganic fluoride concentration [40]. Recently published data for the rivers Rhine and Moselle indicated that maximum concentrations were around ~ 130 μg/L respectively ~ 180 μg/L fluoride [41]. Hence, concentrations at Spree with ~ 200 μg/L were of the same order of magnitude.

Concentrations of sample locations 5, 6, 8, and 10 were in good agreement between the instrumental methods, while for sample locations 1, 2, 3, and 4, CIC_B_ tended to provide higher TF concentrations in comparison to HR-CS-GFMAS_B_. For samples 7 and 9, CIC_B_ tended to provide lower TF concentrations in comparison to HR-CS-GFMAS_B_. Overall, TF concentrations between the two instrumental methods were in the same order of magnitude. Higher TF concentrations provided by CIC_B_ at sample locations 1, 2, 3, and 4 could be due to carry-over effects or cross contamination during the measurements. A higher uncertainty arises due to the instrumental methodology of CIC_B_ while trapping HF, which results from the dilution of the samples by a factor of 20 during the trapping process. Variations of the trapping solution volume could also lead to variations, especially for sample locations 7 and 9, in which lower concentrations were detected for CIC_B_ in comparison to HR-CS-GFMAS_B_. While a comparison of IC and HR-CS-GFMAS for fluoride was published before and results were in better agreement compared to this study [41], combustion-coupled IC might result in higher uncertainty compared to fluoride determination using IC solely. The intended purpose of using CIC rather than IC in this study was to provide a better comparison of total fluorine, because IC analysis provides only the detection of inorganic fluorine species, while CIC provides results for both inorganic and organic fluorine species summarized as TF.

### AOF analysis

**Fig. 5:**
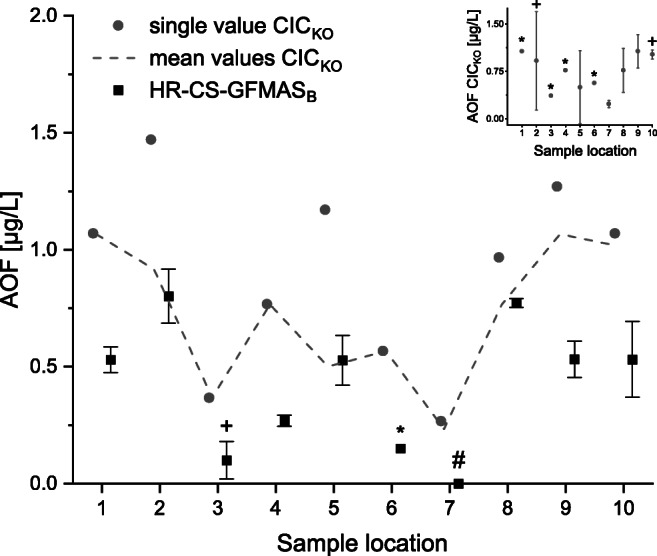
Blank-corrected AOF concentrations in Spree river water samples determined using HR-CS-GFMAS_B_ (square) and CIC_KO_ (circle). The dashed line shows mean values of methodical triplicates of AOF sample analyzed using CIC_KO_. The inlay figure shows AOF concentrations analyzed using CIC_KO_ in methodical triplicate. Error bars are based on *n* = 2 and *n* = 3 ± SD for HR-CS-GFMAS_B_ as well as CIC_KO_ (for the inlay figure in the top right corner); *n* = 1 for CIC_KO_ (without error bars). The mean CRM (diluted 1:1 in a 10 ppm aqueous phosphate solution) fluorine concentration was 63.34 μg/L (acceptable according to the manufacturer). For sample locations, refer to Fig. 2 and Table 1. + = *n* = 2; * = *n* = 1; # = value was set to “0”—due to negative values upon blank correction

During the first two steps of AOF determination (see Adsorption and combustion of organic bound fluorine, Materials and methods section), analytes were adsorbed onto AC and, during combustion, converted and absorbed as HF in a trapping solution. For IC_KO_ measurements, trapping solutions were directly analyzed. The trapping solution of one of the methodic triplicates was collected and analyzed using HR-CS-GFMAS_B_ (see Fig. 5). Furthermore, all AOF values are corrected for the methodic blank value according to von Abercron et al. [34]. Methodic blanks were relatively high compared to the instrumental blank values of both systems, resulting in low corrected analyte values (some were even negative). This means that the determined concentrations are lower than the methodological blank concentrations; hence, concentrations are below the methodological detection limit. Samples were measured in technical triplicates using HR-CS-GFMAS_B_ and results are shown in Fig. 5. The dashed line (Fig. 5) indicates the trend of the methodical triplicate for CIC_KO_ AOF concentrations. Due to the higher standard deviation of the methodical mean values, the overall trend that HR-CS-GFMAS_B_ and CIC_KO_ AOF results were similar along the river Spree in Berlin could not be shown (see inlay Fig. 5). To better compare the results generated using CIC_KO_ and HR-CS-GFMAS_B_, only one sample out of the triplicate (values above LOQ) was analyzed by means of both methods (see Fig. 5).

AOF values determined by HR-CS-GFMAS_B_ varied up to about 0.9 μg/L with the lowest concentration at sampling location 7 and the highest concentration at location 2. Those determined by CIC_KO_ varied between about 0.3 and 1.5 μg/L also with the lowest concentration at sampling location 7 and the highest concentration at location 2. HR-CS-GFMAS_B_ and CIC_KO_ AOF results showed a similar trend along the river Spree in Berlin. The higher AOF concentrations for all samples determined using CIC_KO_ compared to HR-CS-GFMAS_B_ could be due to potential systematic blank value problems. Additionally, all AOF values were nearby the instrumental LOQ of CIC_KO_, which enhanced the uncertainty. Overall, the trends for samples 1–8 were in good agreement between HR-CS-GFMAS_B_ and CIC_KO_, while AOF concentrations determined using CIC_KO_ shifted upwards because of potential systematic blank value problems.

CIC_KO_ AOF concentrations with about 0.3–1.5 μg/L were in good agreement with previously determined AOF concentrations by Wagner et al. with 0.45–2.5 μg/L for WWTPs, surface waters, and ground waters, with concentrations near the LOQ of 0.3 μg/L [36]. Furthermore, Willach et al. determined similar AOF concentrations in the range of 0.88–1.98 μg/L for WWTP effluents and surface waters [33].

### EOF analysis

**Fig. 6:**
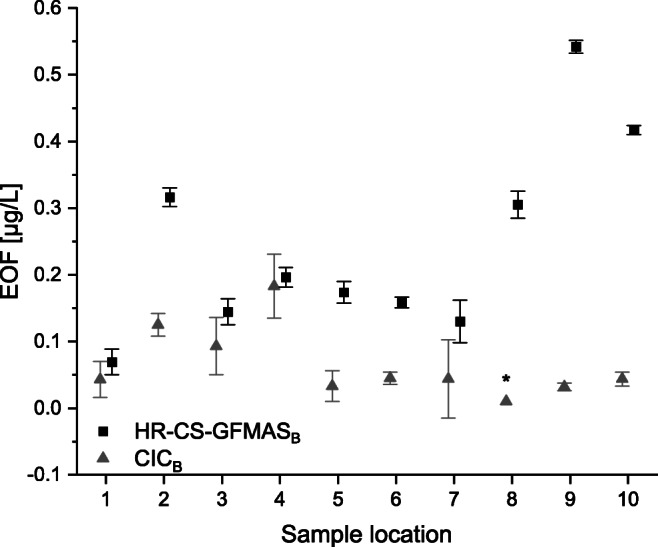
Blank-corrected EOF concentrations in Spree river water samples determined using HR-CS-GFMAS_B_ (square) and CIC_B_ (triangle). Error bars according to *n* = 3 ± SD (methodical triplicate). The mean CRM (diluted 1:1 with methanol) fluorine concentration was 56.4 μg/L (acceptable according to the manufacturer). For sample locations, refer to Fig. 2 and Table 1. *Two samples out of the triplicate were negative after blank correction and hence, values were omitted

Organic fluorine was extracted using HLB-SPE and methanol as eluent in methodic triplicate. The resulting extracts were aliquoted and analyzed by means of HR-CS-GFMAS_B_ and CIC_B_. Results are shown in Fig. 6. In order to assure the same quality level, EOF values were corrected for methodic blank values according to von Abercron et al. [34]. EOF concentrations determined using HR-CS-GFMAS_B_ varied around 0.05–0.55 μg/L, while CIC_B_ EOF concentrations were lower, ranging up to 0.22 μg/L. EOF concentrations between CIC_B_ and HR-CS-GFMAS_B_ were in best agreement at sample locations 1, 3, and 4 with mean differences < 0.05 μg/L. The highest differences were observed at sample locations 8, 9, and 10 with mean differences > 0.3 μg/L. SDs of CIC_B_ EOF triplicates were relatively high compared to HR-CS-GFMAS_B_ as shown in Fig. 6. While CIC_B_ EOF values were relatively consistent, HR-CS-GFMAS_B_ analysis revealed potential EOF hot spots at sample locations 2, 8, 9, and 10.

Although Miyake et al. used WAX-SPE and CIC, the herein published EOF concentrations determined using HLB-SPE and HR-CS-GFMAS_B_ with about 0.05–0.55 μg/L were in good agreement with their EOF values (0.093 μg/L in unpolluted water and 0.562 μg/L at a contaminated site) [40].

EOF values published by Metzger et al. determined using the same SPE method and HR-CS-GFMAS were also in a similar range (0.05–0.30 μg/L for river water samples) [9].

While EOF concentrations determined using HR-CS-GFMAS_B_ and CIC_B_ showed the same trend for sample locations 1 and 3–7, EOF concentrations determined using HR-CS-GFMAS_B_ provided higher concentration values at all sampling locations. The highest differences observed between EOF concentrations determined using HR-CS-GFMAS_B_ and CIC_B_ at sampling locations 2, 8, 9, and 10 could possibly be related to potential loss of volatile HF during CIC_B_ measurements, possibly because of a leaky device or incomplete combustion of the samples. Furthermore, the variation of the trapping solution could be an important factor to consider, which might lead to higher variations of EOF concentrations determined using CIC_B_. The trend of the EOF concentrations determined using HR-CS-GFMAS_B_ cannot be accurately recovered using CIC_B_ probably because the concentrations were near the LOQ of CIC_B_. This could possibly lead to a higher uncertainty for concentrations near the LOQ. Therefore, HR-CS-GFMAS_B_ provided a lower LOQ and was more sensitive especially while conducting EOF analysis. Overall, EOF concentrations were in the same order of magnitude while determined using either HR-CS-GFMAS_B_ or CIC_B_. EOF concentrations presented in this study compared to EOF concentrations described in the literature were also in the same order of magnitude.

### Comparison of AOF determined via CIC and EOF determined via HR-CS-GFMAS

**Fig. 7:**
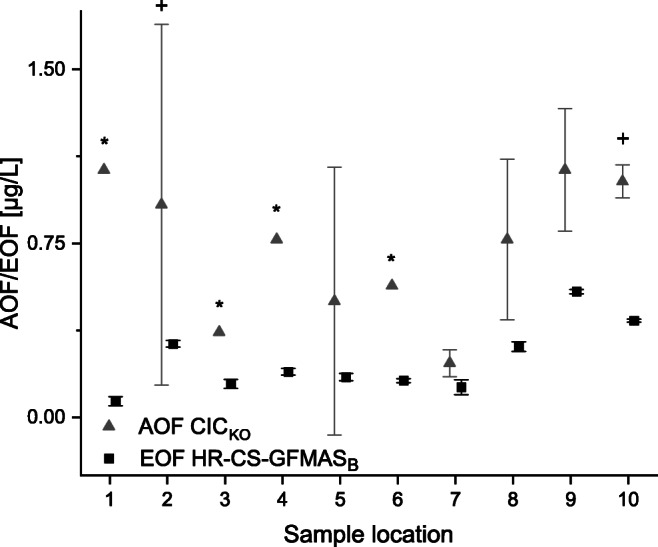
Comparison of EOF determined via HR-CS-GFMAS_B_ (square) and AOF determined via CIC (triangle). Means of methodical triplicates are shown; error bars refer to *n* = 2 and *n* = 3 ± SD (methodical triplicates). The mean CRM (diluted 1:1 with methanol) fluorine concentration was 56.35 μg/L (acceptable according to the manufacturer). For sample locations, refer to Fig. 2 and Table 1. + One sample out of the triplicate was negative after blank correction. * Two samples out of the triplicate were negative after blank correction and hence not taken into account

As shown above, different analytical methods (CIC ↔ HR-CS-GFMAS) are providing comparable results that were in the same order of magnitude for each sum parameter (TF, AOF, as well as EOF). Methodical triplicates of EOF and AOF are compared for the following discussion as well as in Fig. 7.

**Fig. 8:**
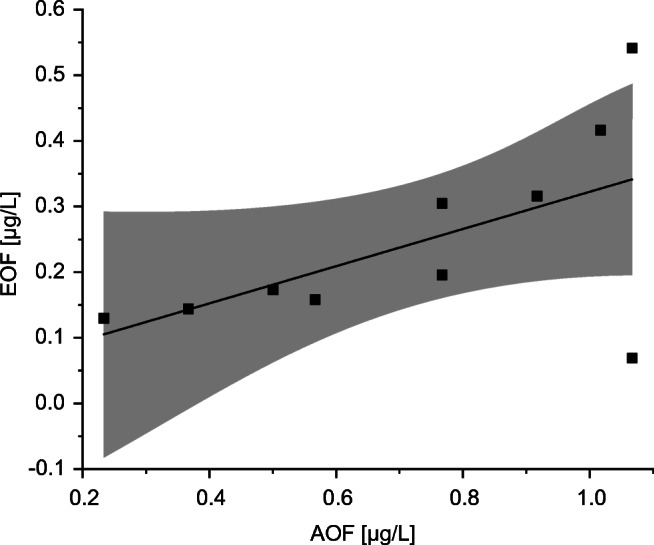
Scatter plot for the comparison of AOF (determined using CIC_KO_) and EOF (determined using HR-CS-GFMAS_B_) with linear regression (black line) and 95% confidence interval (gray area). The equation for the linear regression is y = 0.284 (± 0.143)x + 0.039 (± 0.111)

On average, the quotient between AOF determined via CIC_KO_ and EOF determined via HR-CS-GFMAS_B_ on each sampling location was a factor of about 4, resulting in a slope of about 0.25 in the scatter plot (see Fig. 8). SDs of AOF values were notably higher compared to the SDs of EOF values (average SD values were for AOF 0.54 μg/L and for EOF 0.02 μg/L). A similar trend between AOF and EOF along the sampling locations was observed. The mean values are in best agreement at sampling locations 3 and 7. Highest differences of the mean sum parameter values were observed at sampling locations 2 and 10, which could be due to different selectivity of AOF and EOF, resulting in varying compositions of the measured samples for AOF and EOF. HR-CS-GFMAS_B_ provided noticeably less variation while EOF concentration ranges were similar compared to CIC_KO_ AOF concentrations. Inferring, EOF analysis using HR-CS-GFMAS is less time consuming, more sensitive, and more precise, and for the future prospective, possibly more relevant than AOF analysis conducted by CIC.

By plotting the EOF against the AOF, the scatter plot in Fig. 8 was obtained. The slope of 0.284 (± 0.143) expresses that AOF values are on average systematically higher than EOF values. All values but two (sample locations 2 and 9) were inside the 95% confidence interval. Overall, results were in good agreement and similar trends between determined sum parameters and instrumental approaches could be observed (see Fig. 7).

### Mass balance

Proportionally, AOF determined by CIC_KO_ made up 0.11–0.51% of mean TF (determined using CIC_B_) along the river Spree in Berlin. EOF determined by HR-CS-GFMAS_B_ made up 0.04–0.28% of mean TF (determined using HR-CS-GFMAS_B_) along the river Spree in Berlin. In this study, it could be shown that TF is mainly depended on the inorganic fluoride content. In this context, the results are consistent with the previously published data by Miyake et al. [40]. Despite its small proportion, the OF is crucial due to the extreme environmental persistence and bioaccumulation of PFASs as well as potential severe negative health effects.

## Conclusion

### HR-CS-GFMAS vs. CIC

HR-CS-GFMAS and CIC are both powerful devices in (organically bound) fluorine trace analysis. HR-CS-GFMAS analysis is faster, more sensitive, and more precise compared to CIC respectively IC for fluorine analysis in the low microgram per liter range. When using combustion-coupled IC, the injection of a sample aliquot (~ 200–500 μL) in the sample boat and trapping of HF after combustion in a volume of ~ 10 mL results in high dilution factors (~ 1:20–1:50), which is disadvantageous for detection of low concentrations. Furthermore, the volume of the trapping solution varies, which results in different dilution factors during triplicate measurements of a sample. Additionally, potential loss of volatile HF, possibly because of a leaky device, or incomplete combustion, leads to an underestimation of the fluorine concentration, which can be reduced by means of a basic trapping solution. Consequently, the direct analysis of samples via HR-CS-GFMAS is preferable for EOF determination. Since only < 1% of TF depends on EOF or AOF, the sensitivity and precision in the lower microgram per liter concentration range of the analytical setup is more relevant for risk evaluation. Therefore, the outcome of our comparison study is that HR-CS-GFMAS is beneficial compared to CIC to determine OF.

### AOF vs. EOF

Because of the higher AOF values compared to the EOF values, the AOF seems to represent a higher proportion of the OF. It could be concluded that even lower concentrations of OF are thus better recorded. On the other hand, determined EOF values scattered less and blank value correction had a negligible effect, making the EOF the more precise parameter. The overall higher concentrations of AOF samples could be due to contamination during adsorption of analytes on AC, washing off of inorganic fluoride and the subsequent combustion of AC. Since the determined EOF values are systematically lower than determined AOF values, the OF extraction could be incomplete using HLB phase SPE. According to the systematically lower EOF values, HLB-SPE is indeed more effective than WAX-SPE but further optimization for more accurate determination of OF is needed. With further optimization, EOF might be the superior sum parameter than AOF, but currently, EOF and AOF are equally important in risk evaluation.

The herein presented study is the first comparative study on HR-CS-GFMAS⇔CIC as well as AOF⇔EOF.
