# Correction to: Determination of organically bound fluorine sum parameters in river water samples—comparison of combustion ion chromatography (CIC) and high resolution-continuum source-graphite furnace molecular absorption spectrometry (HR-CS-GFMAS)

**DOI:** 10.1007/s00216-021-03675-z

**Published:** 2021-09-27

**Authors:** Lennart Gehrenkemper, Fabian Simon, Philipp Roesch, Emily Fischer, Marcus von der Au, Jens Pfeifer, Antje Cossmer, Philipp Wittwer, Christian Vogel, Franz-Georg Simon, Björn Meermann

**Affiliations:** 1 Division 1.1 - Inorganic Trace Analysis, Federal Institute for Materials Research and Testing (BAM), Richard-Willstätter-Straße 11, 12489 Berlin, Germany; 2 Division 4.3 - Contaminant Transport and Environmental Technologies, Federal Institute for Materials Research and Testing (BAM), Unter den Eichen 87, 12205 Berlin, Germany; 3 Department G2 - Aquatic Chemistry, Federal Institute of Hydrology (BfG), Am Mainzer Tor 1, 56068 Koblenz, Germany

**Correction to: Analytical and Bioanalytical Chemistry (2021) 413:103–115**

10.1007/s00216-020-03010-y

The article “Determination of organically bound fluorine sum parameters in river water samples—comparison of combustion ion chromatography (CIC) and high resolution-continuum source-graphite furnace molecular absorption spectrometry (HR-CS-GFMAS)”, written by Lennart Gehrenkemper, Fabian Simon, Philipp Roesch, Emily Fischer, Marcus von der Au, Jens Pfeifer, Antje Cossmer, Philipp Wittwer, Christian Vogel, Franz-Georg Simon and Björn Meermann, was originally published Online First without Open Access. After publication in volume 413, issue 1, page 103–115 the author decided to opt for Open Choice and to make the article an Open Access publication. Therefore, the copyright of the article has been changed to © The Author(s) 2021 and the article is forthwith distributed under the terms of the Creative Commons Attribution 4.0 International License, which permits use, sharing, adaptation, distribution and reproduction in any medium or format, as long as you give appropriate credit to the original author(s) and the source, provide a link to the Creative Commons licence, and indicate if changes were made. The images or other third party material in this article are included in the article’s Creative Commons licence, unless indicated otherwise in a credit line to the material. If material is not included in the article’s Creative Commons licence and your intended use is not permitted by statutory regulation or exceeds the permitted use, you will need to obtain permission directly from the copyright holder. To view a copy of this licence, visit http://creativecommons.org/licenses/by/4.0.

The original article has been corrected.

